# Structure of racemic calcium 5-methyl­tetra­hydrofolate trihydrate from synchrotron powder diffraction data and density functional theory

**DOI:** 10.1107/S2056989025010515

**Published:** 2026-01-01

**Authors:** Jacob K. Salazar, James A. Kaduk

**Affiliations:** a Department of Chemistry North Central College, 131 S Loomis St Naperville IL 60540 USA; University of Aberdeen, United Kingdom

**Keywords:** powder diffraction, 5-methyl­tetra­hydro­folate calcium, Metafolin, Rietveld refinement, density functional theory

## Abstract

The crystal structure of the racemic title compound, poly[[(μ-(2*S*)-2-{[4-({[(6*S*)-2-amino-5-methyl-1,4,5,6,7,8-hexahydropteridin-6-yl]methyl}amino)phenyl]formamido}pentanedioato)diaquacalcium(II)] monohydrate], {[Ca(C_20_H_23_N_7_O_6_)(H_2_O)_2_]·H_2_O}_*n*_ (space group *P*2_1_/*c*) has been solved and refined using synchrotron X-ray powder diffraction data and optimized using density functional theory techniques. The structure consists of alternating hydro­philic (Ca/O) and hydro­phobic layers lying parallel to the *bc* plane. An extensive network of O—H⋯O hydrogen bonds link the Ca coordination spheres within the layers. The anions link the Ca^2+^ ions into chains propagating along the *b*-axis direction. N—H⋯O hydrogen bonds link the Ca/O units and the anion layers. The calcium ion is 6-coordinate (distorted octa­hedral), and is isolated. The coordination sphere consists of two water mol­ecules, a chelated carboxyl­ate group, a monodentate carboxyl­ate group, and a carbonyl group. Both the Ca coordination geometry and the extended structure are very different than that of the previously determined enanti­opure calcium l-5-methyl­tetra­hydro­folate trihydrate (Form I).

## Abstract 2

The crystal structure of racemic calcium 5-methyl­tetra­hydro­folate trihydrate has been solved and refined using synchrotron X-ray powder diffraction data, and optimized using density functional theory techniques.

## 1. Chemical context

Levomefolic acid is a metabolite of folic acid (Vitamin B9), and is a major active form of folate found in foods and in the blood circulation. It is transported across membranes, including the blood-brain barrier. It plays an essential role in DNA and protein synthesis. Levomefolate is approved as a food additive, and is designated as a GRAS (generally recognized as safe) compound. Levomefolate plays major roles in the prevention of birth defects and the formation of red blood cells, which prevents anemia, and has been linked to support for cognitive and mental health. Folic acid deficiency can cause neural tube defects in fetuses and irreversible nervous system damage when combined with vitamin B12 deficiency. It is available commercially as a crystalline calcium salt (trade name Metafolin), which has the required stability for use as a supplement (https://www.drugbank.ca/salts/DBSALY001276). The IUPAC name (CAS Registry number 151533-22-1 for the anhydrous salt) is (2*S*)-2-[(4-{[(6*S*)-2-amino-5-methyl-4-oxo-3,6,7,8-tetra­hydro­pteridin-6-yl]methyl­amino}­benzo­yl)amino]­penta­nedioate calcium trihydrate.

Stable crystalline salts of 5-methyl­tetra­hydro­folic acid are disclosed and claimed in US Patent 6,441,168 (Müller *et al.*, 2002 ▸; Eprova AG). The patent includes X-ray powder diffraction data for crystalline Types I–IV of calcium l-5-methyl­tetra­hydro­folate, as well as the amorphous form. The patent includes claims for ‘a water of crystallization of at least one equivalent per equivalent of 5-methyl­tetra­hydro­folic acid’ and ‘≥3 equivalents of water’. Commercial samples are generally described as the trihydrate, although the penta­hydrate is also available commercially.

The powder pattern of a sample of calcium l-5-methyl­tetra­hydro­folate trihydrate, (**I**), did not correspond to those of Type I (Müller *et al.*, 2002 ▸; Kaduk *et al.*, 2023 ▸) or any other reported form of the compound. A laboratory pattern could be indexed on a primitive monoclinic unit cell with *a* = 15.944 (11), *b* = 11.364 (6), *c* = 15.409 (6) Å, *β* = 118.52 (2)°, *V* = 2453.1 (33) Å^3^, and *Z* = 4. The suggested space group by *FOX* (Favre-Nicolin and Černý, 2002 ▸) was *P*2_1_*/c*, which is impossible for a chiral mol­ecule, and led us to conclude that the sample was racemic.
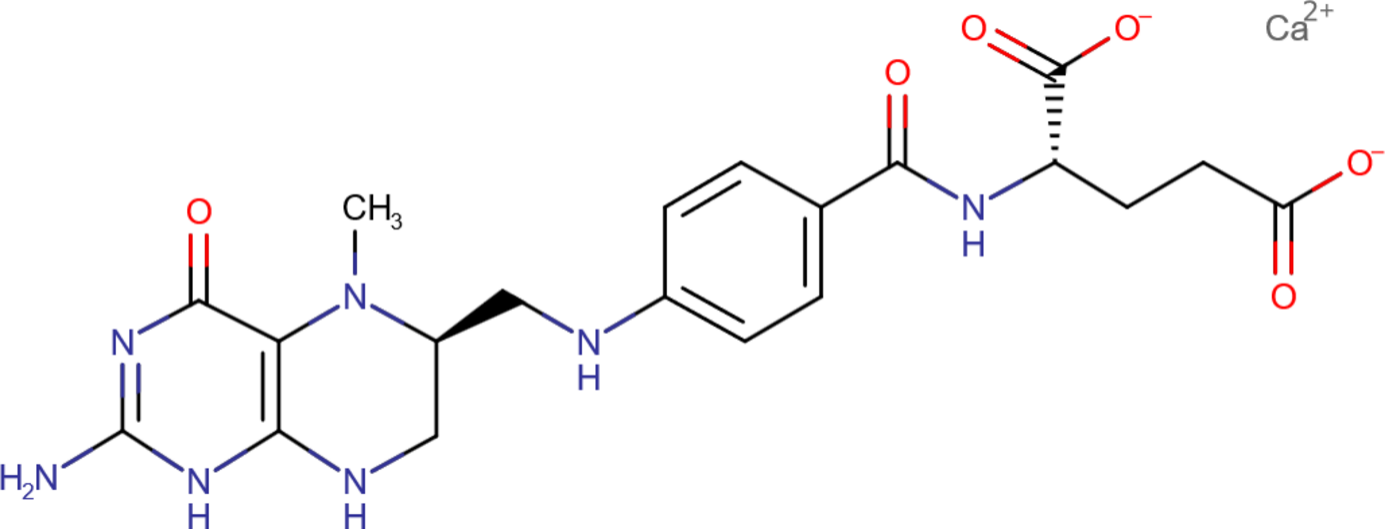


## 2. Structural commentary

**Figure 1:**
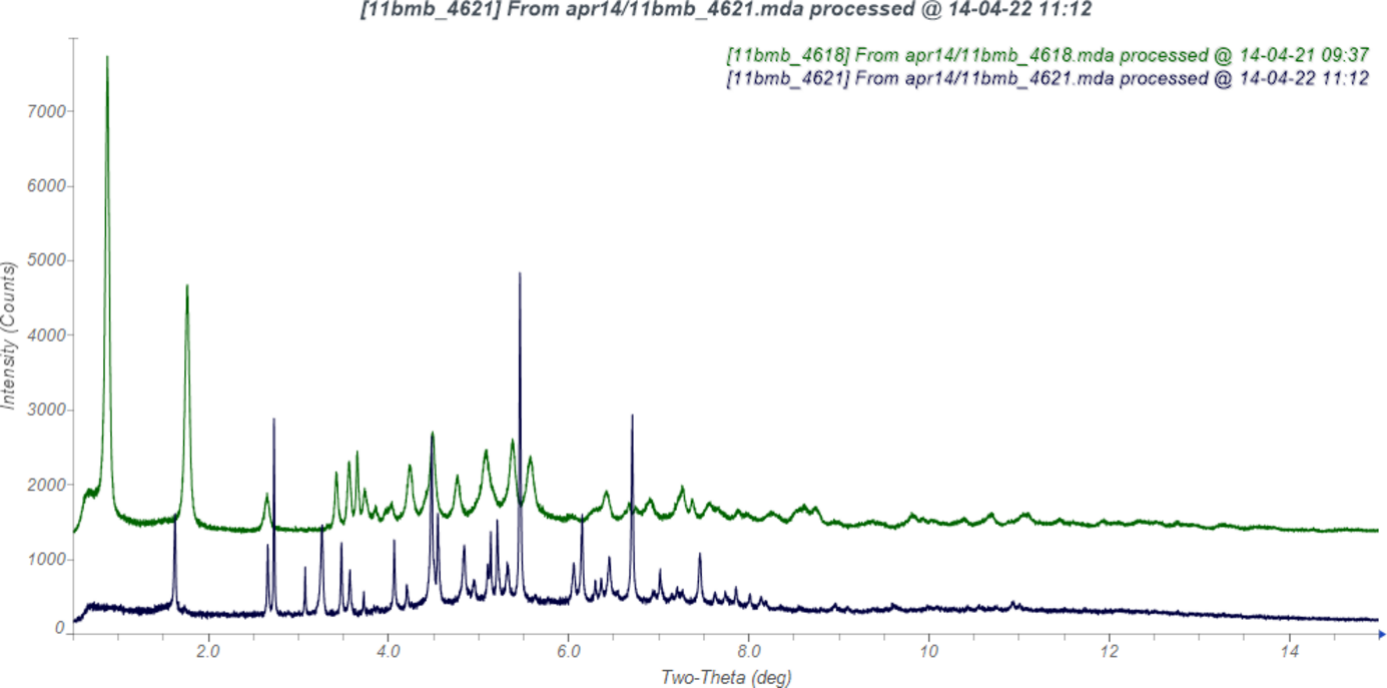
Comparison of the synchrotron diffraction pattern of (**I**) (black) to that of Form I of calcium l-5-methyl­tetra­hydro­folate trihydrate reported by Müller *et al.* (2002 ▸; green). The patent pattern (measured using Cu *K*_α_ radiation) was digitized using *UN-SCAN-IT* (Silk Scientific, 2013 ▸) and converted to the synchrotron wavelength of 0.413691 (2) Å using *JADE Pro* (MDI, 2025 ▸).

The synchrotron powder pattern of (**I**) does not correspond to that of the intended Form I (Fig. 1 ▸, Kaduk *et al.*, 2023 ▸). Solution and refinement of the structure in the centrosymmetric space group *P*2_1_*/c* confirms that it is a racemate instead of the intended enanti­opure material. In the arbitrarily-chosen asymmetric unit, atoms C14 and C25 have *S* configuration, but crystal symmetry generates the other enanti­omer.

**Figure 2:**
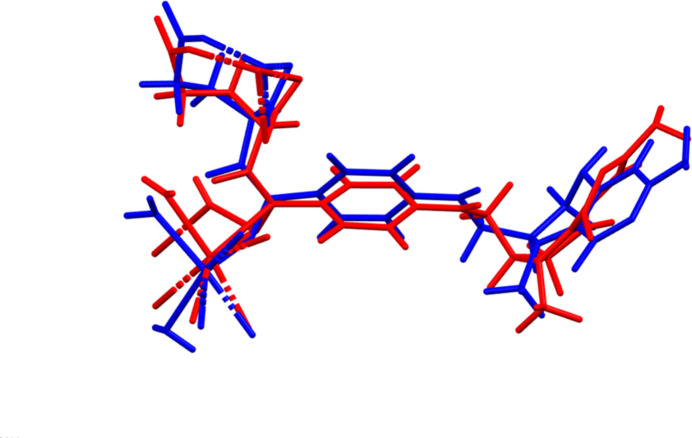
Comparison of the Rietveld-refined structure of (**I**) (red) to the *VASP*-optimized structure (blue). The comparison was generated using the *Mercury* Calculate Mol­ecule Overlay tool; the r.m.s. difference is 1.373 A.

**Figure 3:**
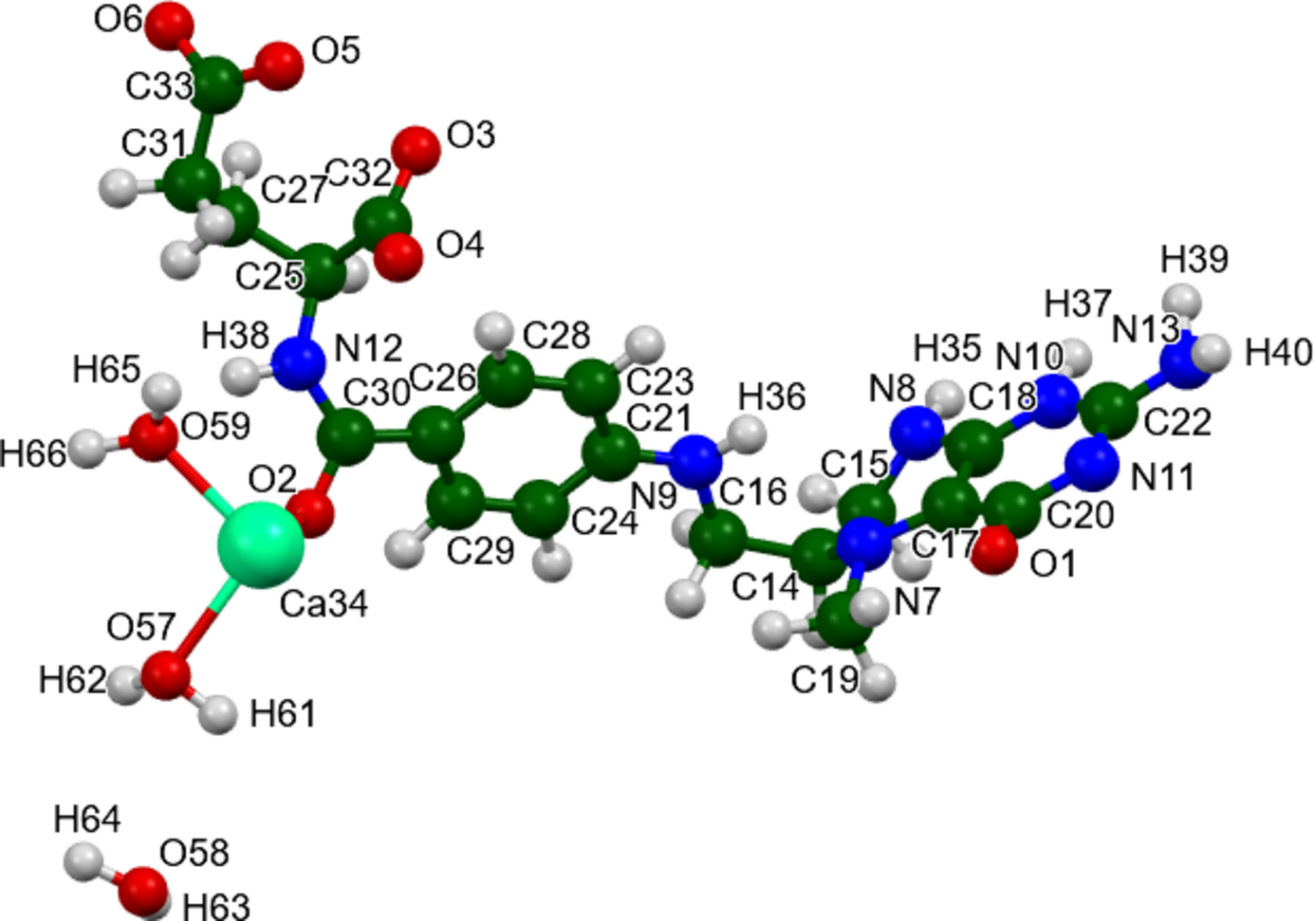
The asymmetric unit of (**I**) with the atom numbering. Image generated using *Mercury* (Macrae *et al.*, 2020 ▸).

The root-mean-square (r.m.s.) difference of the non-H atoms in the Rietveld-refined and VASP-optimized structures of (**I**) calculated using the *Mercury* Calculate/Mol­ecule Overlay tool, is 1.373 Å (Fig. 2 ▸). The agreement is outside the normal range for correct structures (van de Streek & Neumann, 2014 ▸). This large structure, refined using limited data, would be expected to be less accurate than usual. The asymmetric unit is illustrated in Fig. 3 ▸. The refined structure has a close contact between C30 and Ca34, which is relieved on optimization. The *U*_iso_ values of the atoms in the side chain are very large, perhaps indicating disorder. Because the VASP optimization requires an ordered model, we prefer to not attempt to model any disorder. The remaining discussion will emphasize the VASP-optimized structure.

Most of the bond distances, bond angles, and torsion angles for (**I**) fall within the normal ranges indicated by a *Mercury* Mogul Geometry check (Macrae *et al.*, 2020 ▸). The C30—O2 bond length of 1.290 Å [average = 1.240 (15) Å, *Z*-score = 3.4] and the C18—N8 bond of 1.377 Å [average = 1.322 (10) Å, *Z*-score = 5.4] are flagged as unusual. The uncertainties on these averages are relatively low, inflating the *Z*-scores, and only a few similar hits were found. No hits were found for the angles O3—C32—C25, O4—C32—C25, and O5—C33—O6, suggesting that the coordination of the anion to the calcium ion is unusual. The angles C15—C14—N7 [114.0°, average = 108.9 (11)°, *Z*-score = 4.5] and C15—N8—C18 [113.1°, average = 120.1 (4), *Z*-score = 19.1, 2 hits] are flagged as unusual. The uncertainties on both averages are very low, inflating the *Z*-scores. Torsion angles involving rotation about the C30—N12 amide bond lie on the tails of planar distributions. Torsion angles involving rotation about the C14—C16 bond (which reflect the orientation of the fused ring system and the phenyl group) lie on the tails of *gauche/trans* distributions. Both sets of torsion angles are thus slightly unusual.

The Ca^2+^ ion is 6-coordinate (distorted octa­hedral), and is isolated. The bond-valence sum is 2.12. The average deviation from the ideal octa­hedral angles is 14.5 (122)°, and the median deviation is 11.1°. The coordination sphere consists of two water mol­ecules, a chelated carboxyl­ate group O3—C32—O4, a monodentate carboxyl­ate group O5, and a carbonyl group O2. A connectivity search of this coordinate sphere in the Cambridge Structural Database (CSD; Groom *et al.*, 2016 ▸) yielded five hits. CSD refcode FOZDAB (Okamura *et al.*, 2015 ▸) has disordered Ca cations. In the other four [LOBXAE (Das, 2019 ▸), LOMMIJ (Acharya *et al.*, 2000 ▸), PIBSOJ (Cotter *et al.*, 2007 ▸), and WUKZOU (Hong *et al.*, 2020 ▸], one O atom of the chelating carboxyl­ate group also bonds to an adjacent calcium cation. The Ca^2+^ coordination in this structure is unique.

**Figure 4:**
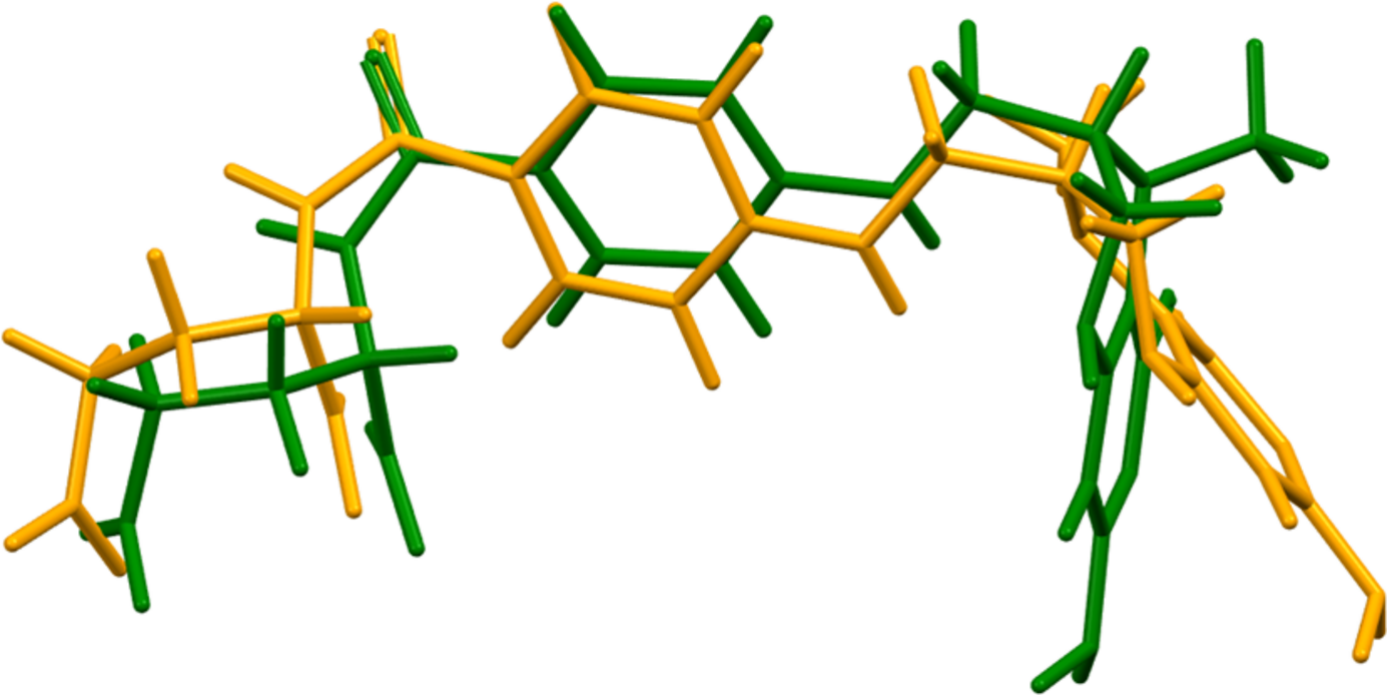
Comparison of the observed structure of the 5-methyl­tetra­hydro­folate anion (orange) to that of a local minimum (green). The r.m.s. difference is 1.284 Å.

Quantum chemical geometry optimization of the isolated 5-methyl­tetra­hydro­folate anion (DFT/B3LYP/6-31G*/water) using *Spartan ’24* (Wavefunction, 2025 ▸) indicated that the observed conformation is 9.6 kJ mol^−1^ higher in energy than a local minimum (Fig. 4 ▸). The r.m.s. Cartesian displacement between the structures is 1.284 Å. The differences are spread throughout the anion, but are most pronounced in the orientation of the tetra­hydro­pteridin ring system. The global minimum-energy conformation (MMFF force field) is 144.7 kJ mol^−1^ lower in energy, and is much more compact; the anion folds upon itself to make the phenyl and tetra­hydro­pteridin ring systems parallel and to form intra­molecular hydrogen bonds. Inter­molecular inter­actions are clearly important to determining the observed solid-state conformation.

**Figure 5:**
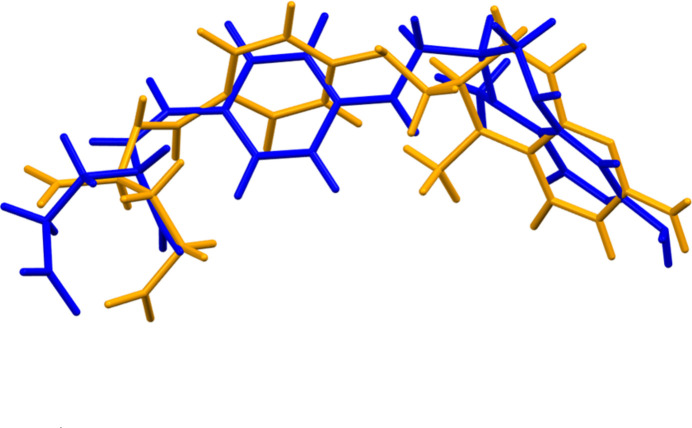
Comparison of the structure of the anion in (**I**) (blue) to that in enanti­opure Type I (orange). The r.m.s. difference is 1.752 Å.

This racemate is 5687.0 kJ mol^−1^ cell^−1^ lower in VASP energy than the previous enanti­opure structure (Kaduk *et al.*, 2023 ▸), even though the unit-cell volumes *V*(rac) = 2532.84 and *V*(old) = 2523.58 Å^3^, and thus the densities (1.446 and 1.453 g cm^−3^, respectively) are similar. The r.m.s. difference is 1.752 Å (Fig. 5 ▸), and the previously determined anion is 67.7 kJ mol^−1^ lower in energy. In this structure each anion coordinates to two Ca^2+^ cations, while in the previous structure each anion coordinates to four Ca^2+^ ions.

In (**I**), the Ca^2+^ ion is 6-coordinate; the coordination sphere consists of one chelated carboxyl group (O3 and O4), one monodentate carboxyl group (O5, which results in a triple chelation to the Ca), a carbonyl group, and two water mol­ecules. In the previous structure the calcium ion is 7-coordinate; the coordination sphere consists of one chelated carboxyl group: O5 and O6, four mondentate carboxyl groups (O3, O4, O5, and O6), and one water mol­ecule. In this structure the Ca^2+^ ion are isolated, while in the previous structure the calcium ions form one-dimensional chains. In this structure there is one isolated water mol­ecule, while in the previous structure there are two.

In this structure there are five independent O—H⋯O hydrogen bonds (Table 1 ▸), while in the previous structure there are only three. Likewise there are four and two N—H⋯O hydrogen bonds, and one and three N—H⋯N hydrogen bonds. respectively. We suspect that the hydrogen bonds are the principal contributors to the lower energy of this structure.

## 3. Supra­molecular features

**Figure 6:**
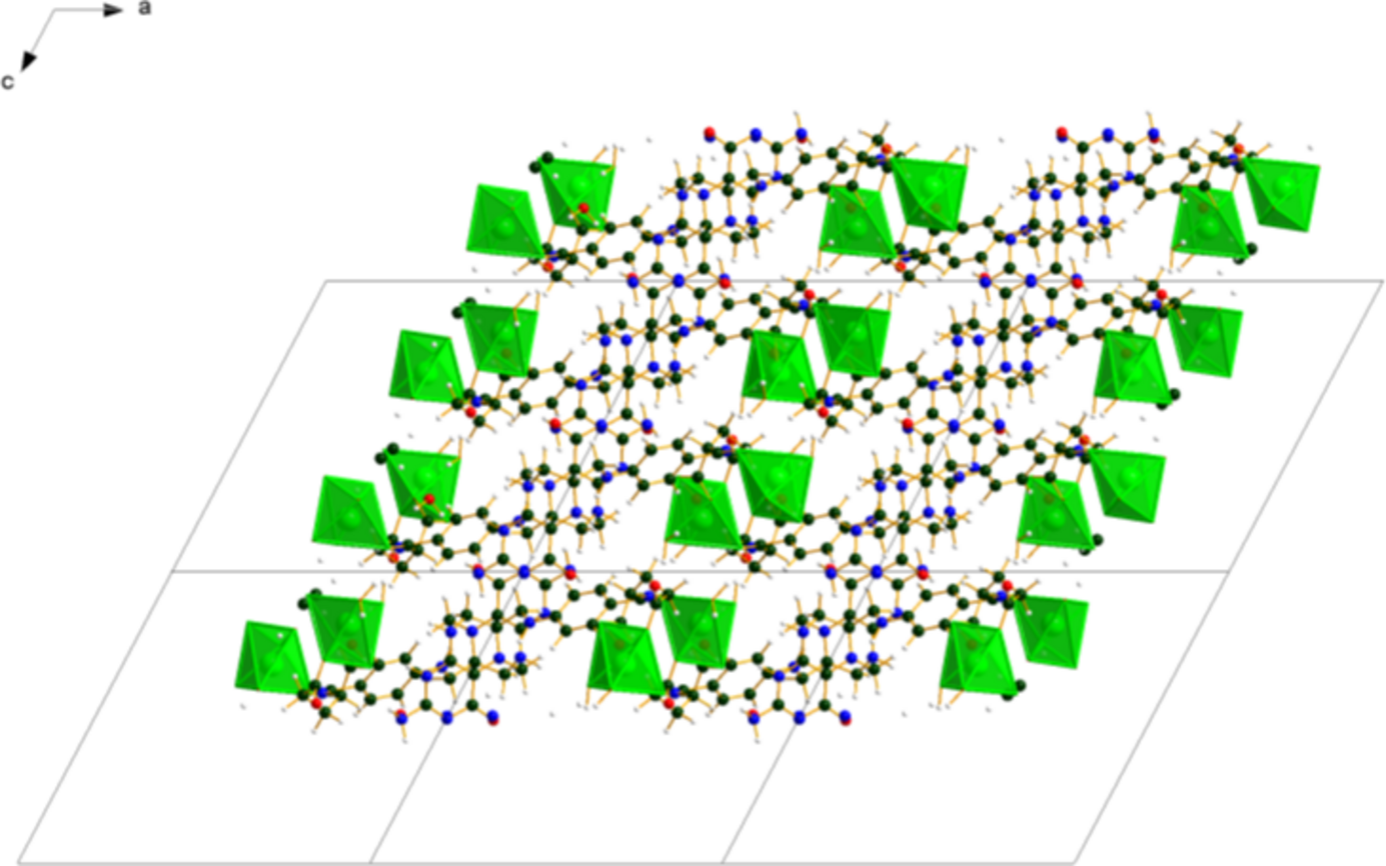
The crystal structure of (**I**) viewed down the *b*-axis direction.

**Figure 7:**
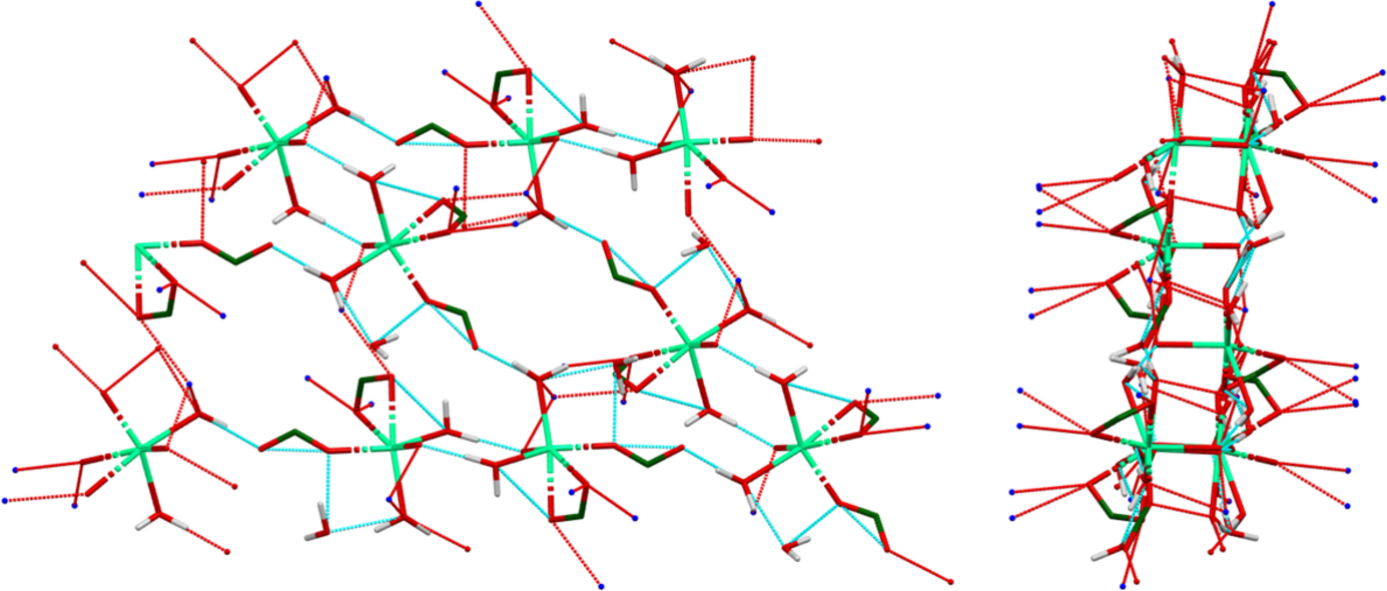
The O—H⋯O hydrogen-bonded layers in (**I**). On the left is a view down the *a*-axis, and on the right is a view down the *c*-axis.

**Figure 8:**
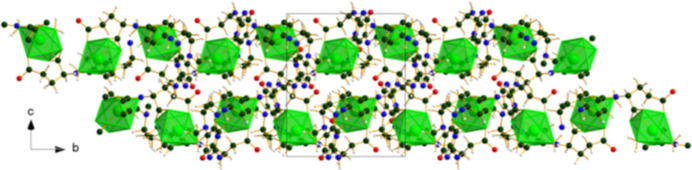
The hydrogen-bonded chains of Ca^2+^ cations propagating along the *b*-axis direction.

**Figure 9:**
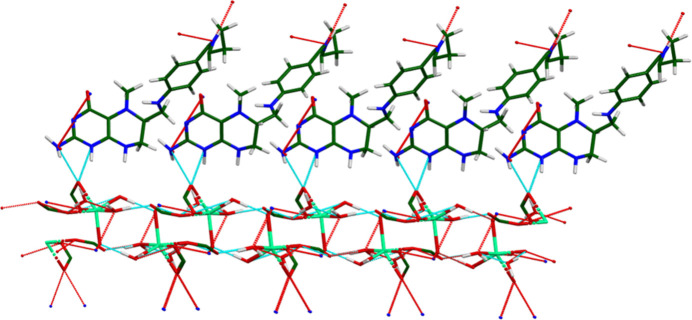
The N—H⋯O hydrogen bonds, which link the hydro­phobic and hydro­philic layers in the extended structure of (**I**).

The crystal structure of (**I**) (Fig. 6 ▸) consists of alternating hydro­philic (Ca/O) and hydro­phobic layers lying parallel to the *bc* plane. An extensive network of O—H⋯O hydrogen bonds (Table 1 ▸) link the Ca coordination spheres within the layers (Fig. 7 ▸). Anions link Ca into chains along the *b*-axis (Fig. 8 ▸) and N10/N13—H⋯O3 hydrogen bonds link the Ca/O and anion layers (Fig. 9 ▸).

**Figure 10:**
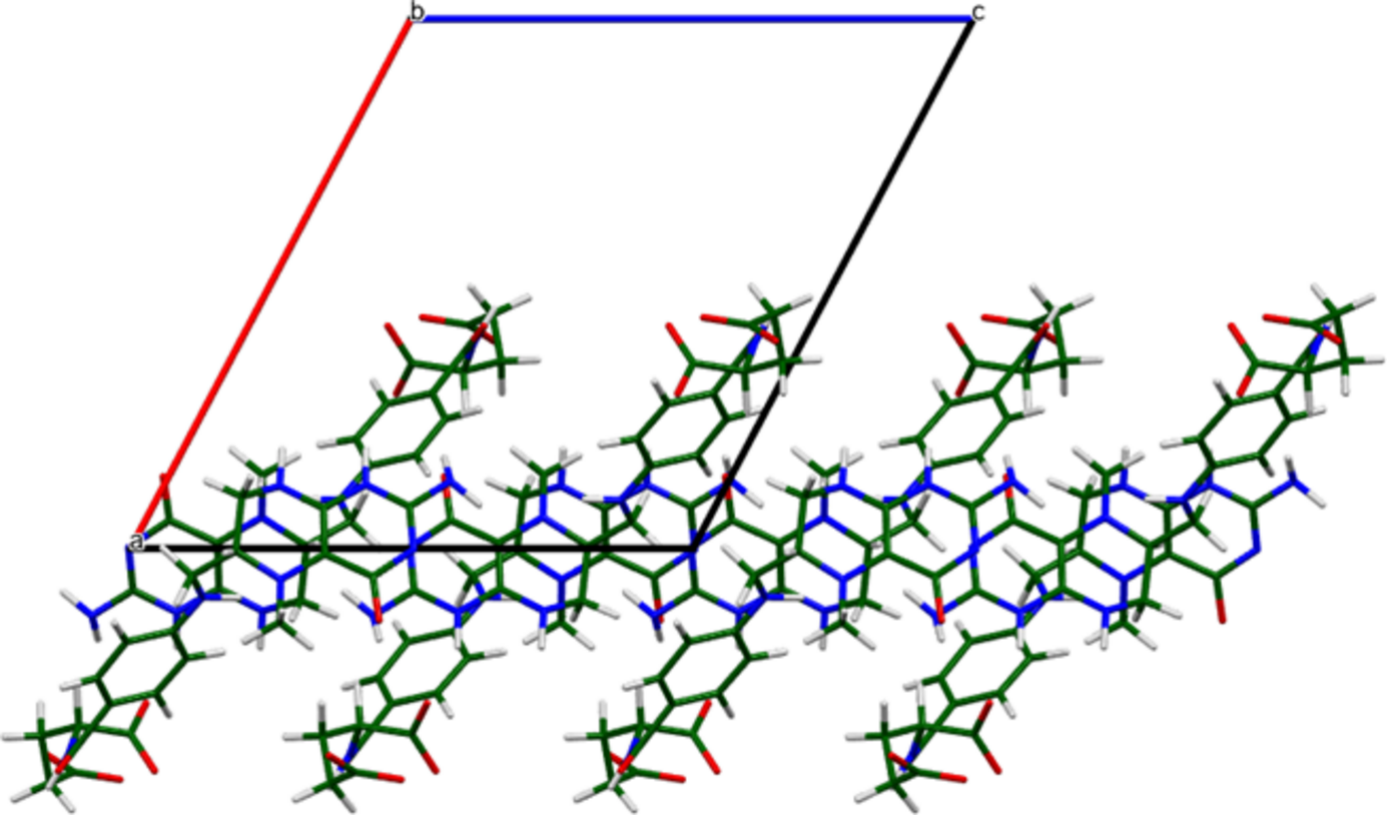
The inter­leaving of the aromatic ring systems in the extended structure of (**I**).

Since both carboxyl groups coordinate to Ca, they make up the outer surface of the ‘hydro­philic’ layer. In the center of these layers, the C/N ring systems inter­leave in a complex manner (Fig. 10 ▸). The closest phen­yl–tetra­hydro­pteridin distance is 4.71 Å (centroids), and the shortest tetra­hydro­pteridin–tetra­hydro­pteridin distance is 6.45 Å. The *Mercury* Aromatics Analyser indicates only weak inter­actions between phenyl rings, with distances > 6.8 Å.

Analysis of the contributions to the total crystal energy of the structure using the Forcite module of *Materials Studio* (Dassault Systèmes, 2024 ▸) indicated that the intra­molecular energy is dominated by angle distortion terms, as expected for a system containing fused rings. The inter­molecular energy is small, and consists mainly of van der Waals attractions. The hydrogen bonds are better discussed using the results of the DFT calculation.

Hydrogen bonds (Table 1 ▸) are important in the crystal structure of (**I**). The coordinated water mol­ecule O57 acts as a donor to an ‘intra­molecular’ carboxyl­ate O4 atom and to the coordinated carbonyl group O2. The coordinated water mol­ecule O59 acts as a donor in two very strong hydrogen bonds to the carboxyl­ate O6 and the free water mol­ecule O58. The energies of the O—H⋯O hydrogen bonds were calculated using the correlation of Rammohan & Kaduk (2018 ▸). The uncoordinated water mol­ecule O58 makes a strong O—H⋯O hydrogen bond to the carboxyl­ate O5, and makes bifurcated O—H⋯(C,C) hydrogen bonds to the phenyl ring atoms C24 and C29, *i.e*., an O—H⋯π link. These are among the most negatively charged C atoms in the anion.

As noted above, the N10/N13—H37/H39⋯O3 hydrogen bonds link the Ca/O and anion layers from the tetra­hydro­pteridin ring systems and one of the carboxyl­ate O atoms. The amide N12 atom makes a strong intra­molecular N—H⋯O hydrogen bond to the coordinated water mol­ecule O59. The ring N8 atom makes a weaker inter­molecular hydrogen bond to the coordinated water mol­ecule O57. The energies of the N—H⋯O hydrogen bonds were calculated using the correlation of Wheatley & Kaduk (2019 ▸). There is an intra­molecular N9—H36⋯N7 hydrogen bond. Several C—H⋯O and two C—H⋯N hydrogen bonds also contribute to the cohesion of the structure.

**Figure 11:**
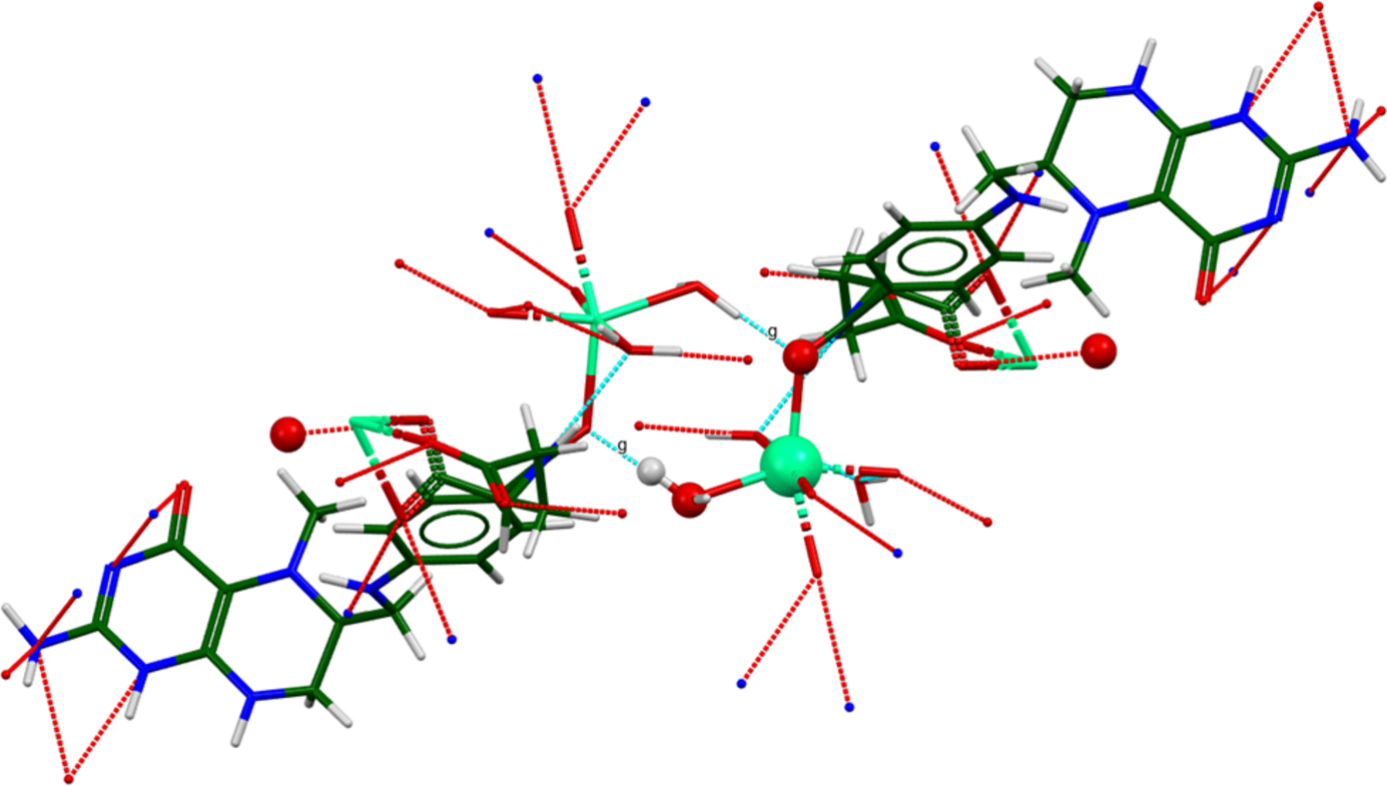
The 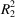
(8) ring pattern that links the Ca^2+^ cations in the extended structure of (**I**).

**Figure 12:**
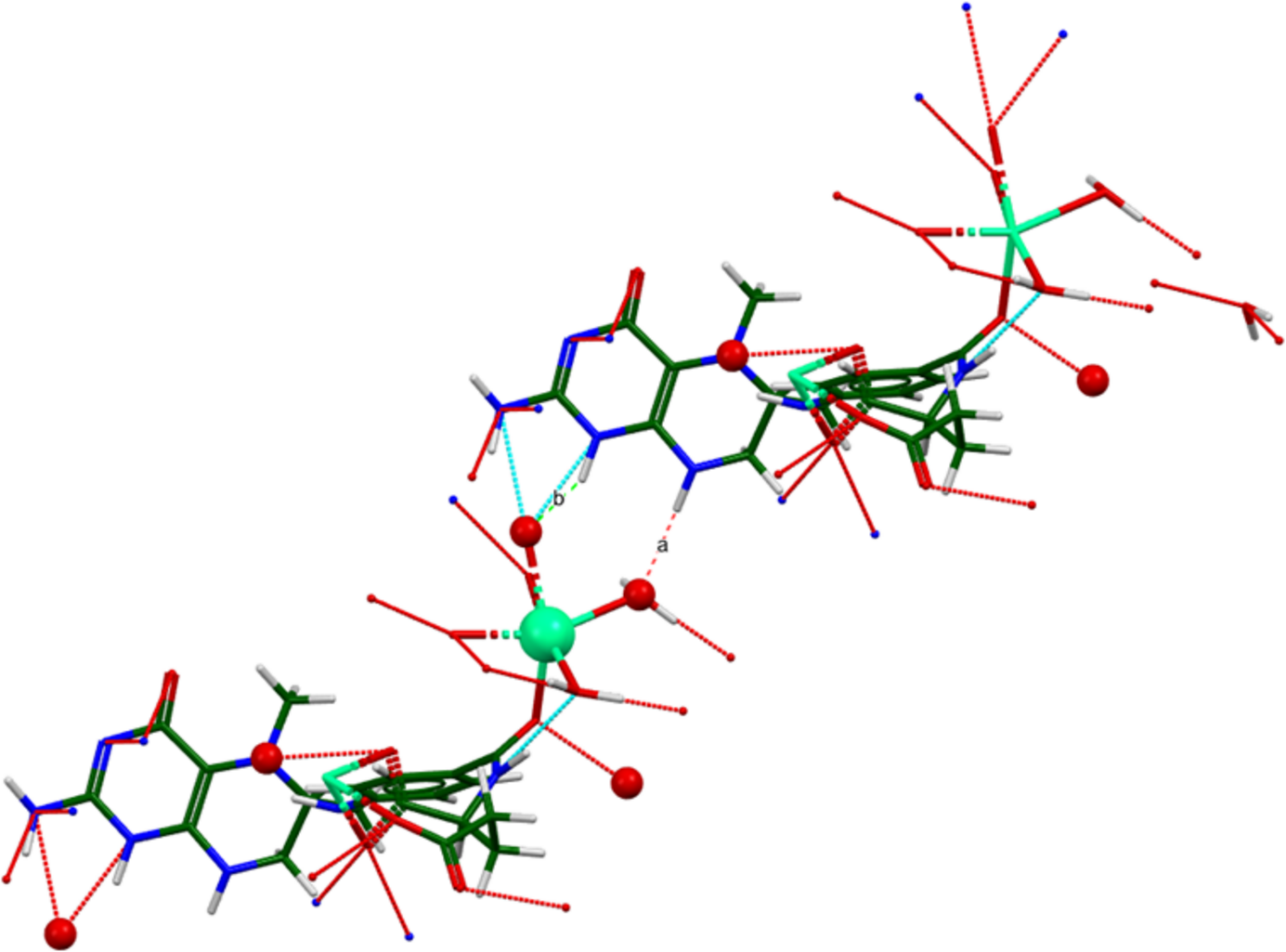
The 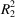
(8) pattern that links the Ca^2+^ cations and the tetra­hydro­pteridin ring system.

Although the Ca coordination spheres are isolated (*i.e*., they do not share corners, edges, or faces), they are linked by a centrosymmetric pair of O57—H62⋯O2 hydrogen bonds between one of the coordinated water mol­ecules and the coordinated carbonyl group. This ring pattern (Fig. 11 ▸) has the graph-set (Etter, 1990 ▸) motif 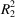
(8). N8 and N10 participate in the same 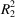
(8) pattern, which links the anion and the Ca coordination sphere (Fig. 12 ▸).

The Bravais–Friedel–Donnay–Harker (Bravais, 1866 ▸; Friedel, 1907 ▸; Donnay & Harker, 1937 ▸) algorithm suggests that we might expect platy morphology for (**I**), with {100} as the major faces. A 4th-order spherical harmonic model was included in the refinement. The texture index was 1.140 (6), indicating that the preferred orientation was significant in this rotated capillary specimen.

## 4. Database survey

A connectivity search of the anion in the Cambridge Structural Database (CSD, Version 2025.2.0; Groom *et al.*, 2016 ▸) yielded no hits; our structure of Type I (Kaduk *et al.*, 2023 ▸) is not yet in the CSD. The powder pattern of Type I is included in the Powder Diffraction File (Kabekkodu *et al.*, 2024 ▸) as entry 00-074-0084. A reduced cell search in the CSD yielded seven hits, but no structures for folate or its derivatives.

## 5. Synthesis and crystallization

The compound stated to be enanti­opure calcium l-5-methyl­tetra­hydro­folate trihydrate (batch #414012519), synthesized by Allastir Private Limited, was supplied by Virtus Pharmaceuticals and used as received.

## 6. Refinement

Crystal data, data collection and structure refinement details are summarized in Table 2 ▸.

The pale-yellow powder was packed into a 1.5 mm diameter Kapton capillary, and rotated during the measurement at ∼50 Hz. The powder pattern was measured at 295 K at beam line 11-BM (Lee *et al.*, 2008 ▸, Wang *et al.*, 2008 ▸, Antao *et al.*, 2008 ▸) of the Advanced Photon Source at Argonne National Laboratory using a wavelength of 0.413691 (2) Å from 0.5–50° 2θ with a step size of 0.001° and a counting time of 0.1 sec step^−1^. The high-resolution powder diffraction data were collected using twelve silicon crystal analyzers that allow for high angular resolution, high precision, and accurate peak positions. A mixture of silicon (NIST SRM 640c) and alumina (NIST SRM 676a) standards (ratio Al_2_O_3_:Si = 2:1 by weight) was used to calibrate the instrument and refine the monochromatic wavelength used in the experiment. The synchrotron pattern was indexed both by *JADE Pro* (MDI, 2025 ▸) and *N-TREOR* (Altomare *et al.*, 2013 ▸) on a similar primitive monoclinic unit cell having *a* = 16.4341, *b* = 11.2869, *c* = 15.4326 Å, *β* = 117.74°, *V* = 2533.6 Å^3^, and *Z* = 4. The space group suggested by both programs was *P*2_1_*/c*, which was confirmed by successful solution and refinement of the structure.

Attempts to solve the structure by Monte Carlo simulated annealing techniques using a Ca atom, a levomefolate anion from the Kaduk *et al.* (2023 ▸) structure, and three O atoms (water mol­ecules) as fragments in several programs were unsuccessful, yielding solutions with severe mol­ecular overlap and/or unreasonable conformations. What turned out to be successful was to use a Ca–levomefolate fragment from the previous structure (chelated through O3 and O4) and three O atoms as fragments in *EXPO2014* (Altomare *et al.*, 2013 ▸). Although this structure had two close inter­molecular contacts, it was used to begin refinement.

In the initial refinement O59 and O60 moved too close to O6 and so were removed from the model, and the N13⋯O3 short contact was still present. A new O59 was placed in the void in the structure; it again moved too close to O6 and was removed from the model. O58 was placed at its original position bound to the Ca, and O59 and O60 were placed in two new voids. After recalculation of the H-atom positions in *Materials Studio* (Dassault Systèmes, 2024 ▸), O60 was too close to the anion, and was relocated in a new void. Refinement of this model yielded a negative occupancy for O59 and O60 too close to the anion. Both were removed from the model.

The arrangement of the Ca cations suggested the possibility that they were bridged by two symmetry-equivalent water mol­ecules. A search of this connectivity in the CSD yielded 44 hits, so this arrangement is not unprecedented. Optimization of this dihydrate model first using the Forcite module of *Materials Studio* and then *VASP* broke one of the bridging Ca—O bonds (yielding two monodentate water mol­ecules) and moved the di­carboxyl­ate side chain of the anion much more than usual, yielded a tris-chelated anion. The poor agreement of the refined and optimized structure, as well as poorer agreement with the diffraction data, led us to abandon this dihydrate model. The trihydrate structure (two coordinated water mol­ecules and one zeolitic) was optimized with *VASP*.

Rietveld refinement of the *VASP*-optimized trihydrate structure was carried out with *GSAS-II* (Toby & Von Dreele, 2013 ▸). Only the 1.0–14.0° portion of the pattern was included in the refinements (*d*_min_ = 1.697 Å). All non-H atom bond distances and angles were subjected to restraints, based on a *Mercury*/Mogul Geometry Check (Sykes *et al.*, 2011 ▸; Bruno *et al.*, 2004 ▸). The Mogul average and standard deviation for each qu­antity were used as the restraint parameters. The phenyl ring was restrained to be planar. The restraints contributed 18.3% to the overall *χ*^2^. The hydrogen atoms were included in calculated positions, which were recalculated during the refinement using *Materials Studio* (Dassault Systèmes, 2024 ▸). The *U*_iso_ values for the heavy atoms were grouped by chemical similarity. For N9 and C16, they refined to slightly negative values, so these were fixed at a reasonable value. The *U*_iso_ values for the hydrogen atoms were fixed at the value of the heavy atoms to which they are attached. The peak profiles were described using the generalized microstrain model (Stephens, 1999 ▸). The background was modeled using a six-term shifted Chebyshev polynomial, with peaks at 1.54, 4.85, and 6.90° to model the scattering from the Kapton capillary and any amorphous component of the sample.

**Figure 13:**
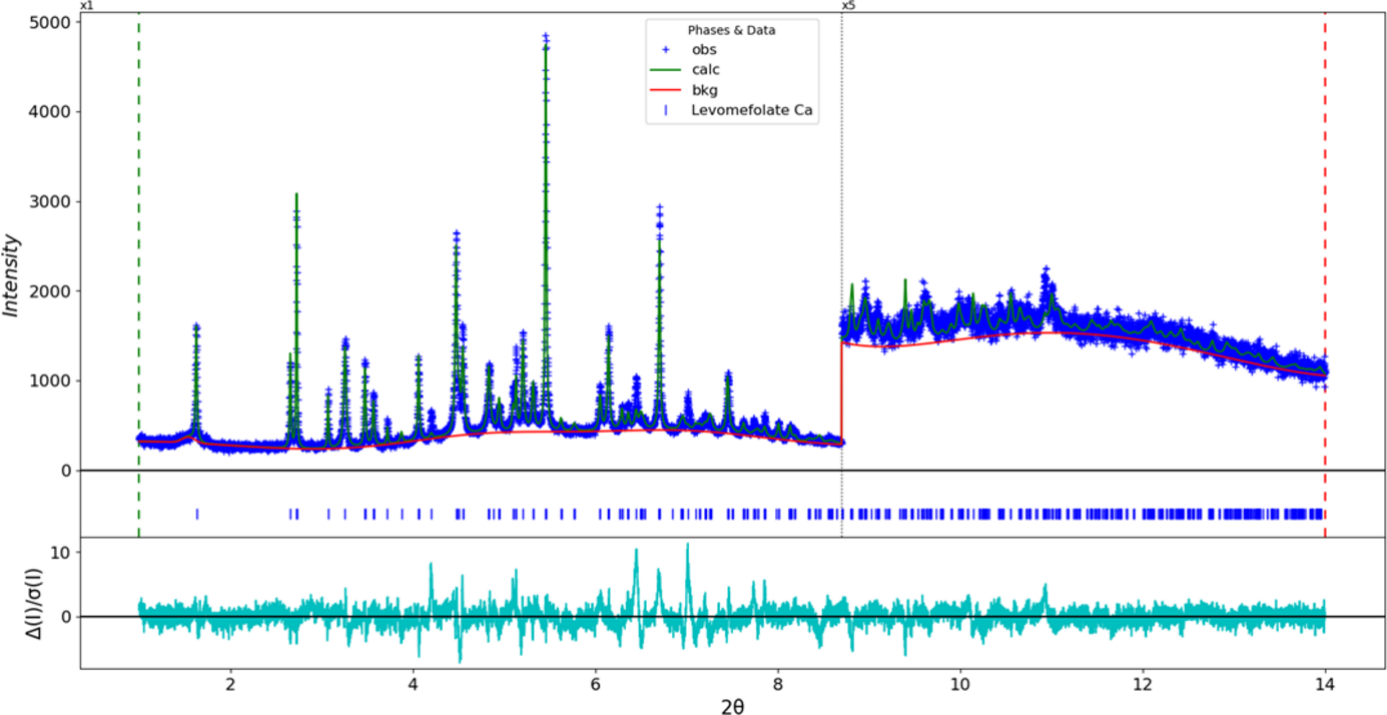
The difference plot for the Rietveld refinement of (**I**). The blue crosses represent the observed data points, and the green line is the calculated pattern. The cyan curve is the normalized error plot, and the red line is the background curve. The blue tick marks indicate the peak positions. The vertical scale has been multiplied by a factor of 5 for 2θ > 8.7°.

The final refinement of 152 variables using 13,002 observations and 106 restraints yielded the residuals *R*_wp_ = 0.089 and GOF = 1.66. The largest peak (1.59 Å from C24) and hole (1.57 Å from C19) in the difference-Fourier map are 0.24 (7) and −0.28 (7) *e* Å^−3^, respectively. The final Rietveld plot is shown in Fig. 13 ▸. The largest features in the normalized error plot are the intensities of the 320 peak at 6.45° and the 13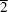 peak at 7.02°.

The crystal structure of racemic calcium 5-methyl­tetra­hydro­folate trihydrate was optimized (fixed experimental unit cell) with density functional techniques using *VASP* (Kresse & Furthmüller, 1996 ▸) through the *MedeA* graphical inter­face (Materials Design, 2024 ▸). The calculation was carried out on 32 cores of a 144-core (768 Gb memory) HPE Superdome Flex 280 Linux server at North Central College. The calculation used the GGA-PBE functional, a plane wave cutoff energy of 400.0 eV, and a *k*-point spacing of 0.5 Å^−1^ leading to a 1 × 2 × 1 mesh, and took ∼26.2 h. Single-point density functional calculations (fixed experimental cell) and population analysis were carried out using *CRYSTAL23* (Erba *et al.*, 2023 ▸). The basis sets for the H, C, N, and O atoms in the calculation were those of Gatti *et al.* (1994 ▸), and that for Ca was that of Peintinger *et al.* (2013 ▸). The calculations were run on a 3.5 GHz PC using 8 *k*-points and the B3LYP functional, and took about 4.0 h.

## Supplementary Material

Crystal structure: contains datablock(s) I, I_VASP. DOI: 10.1107/S2056989025010515/hb8169sup1.cif

CCDC references: 2505413, 2505412

Additional supporting information:  crystallographic information; 3D view; checkCIF report

## Figures and Tables

**Table 1:** Hydrogen-bond geometry (Å, °) for I_VASP[Chem scheme1]

*D*—H⋯*A*	*D*—H	H⋯*A*	*D*⋯*A*	*D*—H⋯*A*
N8—H35⋯O57^i^	1.02	2.48	3.490	169
N10—H37⋯O3^ii^	1.03	1.85	2.805	152
N12—H38⋯O59	1.03	2.00	2.991	160
N13—H39⋯O3^ii^	1.03	2.21	2.928	125
N13—H39⋯O1^iii^	1.03	2.34	3.086	128
O57—H61⋯O4^iv^	0.98	2.24	2.888	122
O57—H62⋯O2^v^	1.01	1.62	2.625	176
O58—H64⋯O5^vi^	1.00	1.81	2.749	156
O59—H65⋯O58^v^	1.00	1.66	2.588	151
O59—H66⋯O6^vii^	1.04	1.49	2.525	170
C16—H45⋯O1^viii^	1.10	2.47	3.349	136
C19—H48⋯O1	1.09	2.06	2.791	121
C24—H50⋯O1^viii^	1.09	2.14	3.157	154
C25—H51⋯N13^ix^	1.10	2.22	3.120	137
C28—H54⋯O4	1.09	2.28	3.263	150
C31—H60⋯O58^v^	1.10	2.37	3.446	165

Symmetry codes: (i) 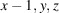; (ii) 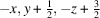; (iii) 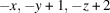; (iv) 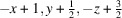; (v) 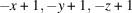; (vi) 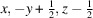; (vii) 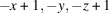; (viii) 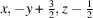; (ix) 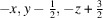.

**Table 2:** Experimental details

	(I)
Crystal data	
Chemical formula	[Ca(C_20_H_23_N_7_O_6_)(H_2_O)_2_]·H_2_O
*M*_r_	551.57
Crystal system, space group	Monoclinic, *P*2_1_/*c*
Temperature (K)	295
*a*, *b*, *c* (Å)	16.462 (2), 11.2800 (4), 15.431 (2)
β (°)	117.854 (4)
*V* (Å^3^)	2533.30 (9)
*Z*	4
Radiation type	Synchotron, λ = 0.41369 Å
μ (mm^−1^)	0.02
Specimen shape, size (mm)	Cylinder, 3 × 1.5

Data collection	
Diffractometer	11-BM APS
Specimen mounting	Kapton capillary
Data collection mode	Transmission
Scan method	Step
2θ values (°)	2θ_min_ = 0.500 2θ_max_ = 49.989 2θ_step_ = 0.001

Refinement	
*R* factors and goodness of fit	*R*_p_ = 0.061, *R*_wp_ = 0.081, *R*_exp_ = 0.054, *R*(*F*^2^) = 0.20984, χ^2^ = 2.766
No. of parameters	152
No. of restraints	106
(Δ/σ)_max_	0.562

Computer programs: *GSAS-II* (Toby & Von Dreele, 2013 ▸), *DIAMOND* (Putz & Brandenburg, 2025 ▸) and *Mercury* (Macrae *et al.*, 2020 ▸).

## supplementary crystallographic information

### (I) Crystal data

**Table:**

C_20_H_29_CaN_7_O_9_	*V* = 2533.30 (9) Å^3^
*M**_r_* = 551.57	*Z* = 4
Monoclinic, *P*2_1_/*c*	*D*_x_ = 1.446 Mg m^−^^3^
*a* = 16.462 (2) Å	Synchotron radiation, λ = 0.41369 Å
*b* = 11.2800 (4) Å	µ = 0.02 mm^−^^1^
*c* = 15.431 (2) Å	*T* = 295 K
β = 117.854 (4)°	cylinder, 3 × 1.5 mm

### (I) Data collection

**Table:**

11-BM APS diffractometer	Scan method: step
Specimen mounting: Kapton capillary	2θ_min_ = 0.500°, 2θ_max_ = 49.989°, 2θ_step_ = 0.001°
Data collection mode: transmission	

### (I) Refinement

**Table:**

Least-squares matrix: full	152 parameters
*R*_p_ = 0.061	106 restraints
*R*_wp_ = 0.080	28 constraints
*R*_exp_ = 0.054	Weighting scheme based on measured s.u.'s
*R*(*F*^2^) = 0.20984	(Δ/σ)_max_ = 0.562
49497 data points	Background function: Background function: "chebyschev-1" function with 6 terms: 221(3), 6.1(20), 61(5), -88(3), -17.5(18), 28.1(21), Background peak parameters: pos, int, sig, gam: 4.85(6), 7.5(5)e4, 9.8(6)e3, 0.100, 6.90(6), 7.7(6)e4, 1.04(7)e4, 0.100, 1.542(8), 1.17(13)e3, 49(10), 0.100,
Profile function: Finger-Cox-Jephcoat function parameters U, V, W, X, Y, SH/L: peak variance(Gauss) = Utan(Th)^2^+Vtan(Th)+W: peak HW(Lorentz) = X/cos(Th)+Ytan(Th); SH/L = S/L+H/L U, V, W in (centideg)^2^, X & Y in centideg 1.163, -0.126, 0.063, 0.000, 0.000, 0.002,	Preferred orientation correction: Simple spherical harmonic correction Order = 4 Coefficients: 0:0:C(2,-2) = 0.097(18); 0:0:C(2,0) = -0.310(20); 0:0:C(2,2) = -0.224(25); 0:0:C(4,-4) = -0.788(25); 0:0:C(4,-2) = 0.152(21); 0:0:C(4,0) = -0.565(24); 0:0:C(4,2) = -0.093(29); 0:0:C(4,4) = -0.106(28)

### (I) Fractional atomic coordinates and isotropic or equivalent isotropic displacement parameters (Å^2^)

**Table:**

	*x*	*y*	*z*	*U*_iso_*/*U*_eq_	
O1	0.0612 (13)	0.654 (2)	0.9996 (8)	0.086 (7)*	
O2	0.3927 (14)	0.4819 (15)	0.5608 (15)	0.353 (12)*	
O3	0.3201 (13)	0.0703 (17)	0.7151 (16)	0.353 (12)*	
O4	0.4513 (14)	0.1719 (17)	0.7822 (12)	0.353 (12)*	
O5	0.4693 (18)	−0.095 (2)	0.6667 (14)	0.353 (12)*	
O6	0.420 (3)	−0.1450 (18)	0.5111 (16)	0.353 (12)*	
N7	0.0588 (8)	0.7359 (10)	0.8125 (8)	0.086 (7)*	
N8	−0.1186 (9)	0.7459 (15)	0.6733 (10)	0.086 (7)*	
N9	0.1252 (12)	0.5511 (15)	0.7411 (13)	0.0800*	
N10	−0.1617 (7)	0.6105 (17)	0.7545 (12)	0.086 (7)*	
N11	−0.0516 (11)	0.5285 (12)	0.9040 (11)	0.086 (7)*	
N12	0.4084 (13)	0.2978 (14)	0.6214 (18)	0.353 (12)*	
N13	−0.2048 (13)	0.481 (2)	0.8413 (19)	0.086 (7)*	
C14	0.0421 (9)	0.7403 (15)	0.7101 (9)	0.086 (7)*	
C15	−0.0510 (12)	0.7969 (18)	0.6481 (12)	0.086 (7)*	
C16	0.0457 (10)	0.618 (2)	0.6735 (11)	0.0800*	
C17	−0.0086 (7)	0.6796 (14)	0.8253 (7)	0.086 (7)*	
C18	−0.0929 (7)	0.6668 (11)	0.7448 (7)	0.086 (7)*	
C19	0.1103 (19)	0.836 (2)	0.8741 (16)	0.086 (7)*	
C20	0.0119 (10)	0.6152 (14)	0.9164 (7)	0.086 (7)*	
C21	0.1867 (10)	0.514 (3)	0.7123 (14)	0.307 (14)*	
C22	−0.1376 (10)	0.540 (2)	0.8344 (15)	0.086 (7)*	
C23	0.2803 (10)	0.519 (6)	0.7755 (17)	0.307 (14)*	
C24	0.1562 (12)	0.475 (5)	0.6169 (15)	0.307 (14)*	
C25	0.3492 (13)	0.1977 (15)	0.6108 (13)	0.353 (12)*	
C26	0.3120 (13)	0.442 (2)	0.6485 (16)	0.307 (14)*	
C27	0.345 (2)	0.109 (2)	0.5327 (12)	0.353 (12)*	
C28	0.3422 (11)	0.480 (5)	0.7446 (16)	0.307 (14)*	
C29	0.2183 (14)	0.443 (5)	0.5847 (18)	0.307 (14)*	
C30	0.3781 (17)	0.4084 (14)	0.6120 (18)	0.353 (12)*	
C31	0.439 (2)	0.0578 (16)	0.552 (2)	0.353 (12)*	
C32	0.3787 (14)	0.1371 (18)	0.7102 (12)	0.353 (12)*	
C33	0.4518 (16)	−0.0721 (16)	0.5796 (14)	0.353 (12)*	
H35	−0.19170	0.77330	0.63258	0.0860*	
H36	0.13503	0.53082	0.81578	0.0800*	
H37	−0.23431	0.62330	0.69841	0.0860*	
H38	0.48079	0.28272	0.63774	0.3530*	
H39	−0.26479	0.47245	0.76741	0.0860*	
H40	−0.22538	0.53023	0.89017	0.0860*	
H41	0.09738	0.79731	0.70599	0.0860*	
H42	−0.04601	0.89647	0.66233	0.0860*	
H43	−0.07304	0.78004	0.56729	0.0860*	
H44	−0.01902	0.56744	0.66078	0.0800*	
H45	0.04698	0.62727	0.60060	0.0800*	
H46	0.06109	0.89824	0.88506	0.0860*	
H47	0.14541	0.88674	0.83676	0.0860*	
H48	0.16439	0.80226	0.94830	0.0860*	
H49	0.30626	0.55502	0.85297	0.3070*	
H50	0.07940	0.46907	0.56474	0.3070*	
H51	0.27683	0.23374	0.58496	0.3530*	
H52	0.31251	0.15519	0.45838	0.3530*	
H53	0.29985	0.03045	0.52912	0.3530*	
H54	0.41885	0.47878	0.79795	0.3070*	
H55	0.19196	0.41720	0.50459	0.3070*	
H56	0.44720	0.06720	0.48279	0.3530*	
H60	0.49554	0.11077	0.61325	0.3530*	
Ca34	0.5495 (10)	0.4654 (11)	0.6731 (10)	0.339 (13)*	
O57	0.6087 (18)	0.5830 (18)	0.5584 (19)	0.0879*	
H61	0.60810	0.55340	0.50400	0.0879*	
H62	0.61710	0.65090	0.59210	0.0879*	
O59	0.5736 (15)	0.2707 (16)	0.6397 (16)	0.0879*	
H65	0.59890	0.25060	0.70350	0.0879*	
H66	0.57580	0.22990	0.59080	0.0879*	
O58	0.356 (3)	0.785 (3)	0.182 (3)	0.0879*	
H63	0.31080	0.83930	0.16760	0.0879*	
H64	0.39050	0.72700	0.17760	0.0879*	

### (I) Geometric parameters (Å, º)

**Table:**

O1—C20	1.235 (3)	C27—C31	1.545 (5)
O2—C30	1.243 (2)	C27—H52	1.14 (2)
O2—Ca34	2.351 (16)	C27—H53	1.14 (3)
O2—H61^i^	1.07 (2)	C28—C23	1.386 (4)
O3—C32	1.254 (4)	C28—C26	1.393 (3)
O3—H40^ii^	1.71 (2)	C28—H54	1.140 (15)
O3—Ca34^iii^	2.350 (14)	C29—C24	1.378 (4)
O4—C32	1.256 (4)	C29—C26	1.389 (3)
O4—Ca34^iii^	2.431 (14)	C29—H55	1.139 (15)
O5—C33	1.263 (4)	C30—O2	1.243 (2)
O5—Ca34^iii^	2.718 (15)	C30—N12	1.326 (5)
O6—C33	1.246 (4)	C30—C26	1.489 (3)
N7—C14	1.473 (2)	C30—Ca34	2.60 (2)
N7—C17	1.370 (4)	C31—C27	1.545 (5)
N7—C19	1.468 (3)	C31—C33	1.514 (3)
N8—C15	1.4582 (17)	C31—H56	1.14 (2)
N8—C18	1.3248 (17)	C31—H60	1.14 (3)
N8—H35	1.110 (11)	C32—O3	1.254 (4)
N9—C16	1.445 (3)	C32—O4	1.256 (4)
N9—C21	1.347 (5)	C32—C25	1.536 (3)
N9—H36	1.110 (16)	C32—Ca34^iii^	2.530 (18)
N10—C18	1.368 (3)	C33—O5	1.263 (4)
N10—C22	1.364 (4)	C33—O6	1.246 (4)
N10—H37	1.110 (11)	C33—C31	1.514 (3)
N11—C20	1.379 (3)	H35—N8	1.110 (11)
N11—C22	1.323 (4)	H36—N9	1.110 (16)
N12—C25	1.450 (3)	H37—N10	1.110 (11)
N12—C30	1.326 (5)	H38—N12	1.110 (17)
N12—H38	1.110 (17)	H38—O59	1.52 (2)
N13—C22	1.336 (4)	H39—N13	1.11 (2)
N13—H39	1.11 (2)	H40—O3^iv^	1.71 (2)
N13—H40	1.11 (3)	H40—N13	1.11 (3)
C14—N7	1.473 (2)	H41—C14	1.141 (11)
C14—C15	1.5164 (18)	H42—C15	1.14 (2)
C14—C16	1.506 (6)	H43—C15	1.140 (18)
C14—H41	1.141 (11)	H44—C16	1.140 (19)
C15—N8	1.4582 (17)	H45—C16	1.140 (18)
C15—C14	1.5164 (18)	H46—C19	1.14 (3)
C15—H42	1.14 (2)	H47—C19	1.14 (2)
C15—H43	1.140 (18)	H48—C19	1.14 (3)
C16—N9	1.445 (3)	H49—C23	1.140 (15)
C16—C14	1.506 (6)	H50—C24	1.140 (16)
C16—H44	1.140 (19)	H51—C25	1.14 (2)
C16—H45	1.140 (18)	H52—C27	1.14 (2)
C17—N7	1.370 (4)	H53—C27	1.14 (3)
C17—C18	1.370 (5)	H54—C28	1.140 (15)
C17—C20	1.474 (6)	H55—C29	1.139 (15)
C18—N8	1.3248 (17)	H56—C31	1.14 (2)
C18—N10	1.368 (3)	H60—C31	1.14 (3)
C18—C17	1.370 (5)	Ca34—O2	2.351 (16)
C19—N7	1.468 (3)	Ca34—O3^v^	2.350 (14)
C19—H46	1.14 (3)	Ca34—O4^v^	2.431 (14)
C19—H47	1.14 (2)	Ca34—O5^v^	2.718 (15)
C19—H48	1.14 (3)	Ca34—C30	2.60 (2)
C20—O1	1.235 (3)	Ca34—C32^v^	2.530 (18)
C20—N11	1.379 (3)	Ca34—O57	2.729 (14)
C20—C17	1.474 (6)	Ca34—O59	2.332 (14)
C21—N9	1.347 (5)	O57—Ca34	2.729 (14)
C21—C23	1.387 (3)	O57—H61	0.90 (2)
C21—C24	1.388 (3)	O57—H62	0.90 (2)
C22—N10	1.364 (4)	H61—O2^i^	1.07 (2)
C22—N11	1.323 (4)	H61—O57	0.90 (2)
C22—N13	1.336 (4)	H62—O57	0.90 (2)
C23—C21	1.387 (3)	O59—H38	1.52 (2)
C23—C28	1.386 (4)	O59—Ca34	2.332 (14)
C23—H49	1.140 (15)	O59—H65	0.90 (2)
C24—C21	1.388 (3)	O59—H66	0.900 (19)
C24—C29	1.378 (4)	H65—O59	0.90 (2)
C24—H50	1.140 (16)	H65—H66	1.6141
C25—N12	1.450 (3)	H65—O58^i^	1.61 (3)
C25—C27	1.543 (4)	H66—O59	0.900 (19)
C25—C32	1.536 (3)	H66—H65	1.6141
C25—H51	1.14 (2)	O58—H65^i^	1.61 (3)
C26—C28	1.393 (3)	O58—H63	0.90 (3)
C26—C29	1.389 (3)	O58—H64	0.90 (3)
C26—C30	1.489 (3)	H63—O58	0.90 (3)
C27—C25	1.543 (4)	H64—O58	0.90 (3)
			
C30—O2—Ca34	87.1 (12)	N10—C22—N11	122.6 (2)
C30—O2—H61^i^	115.0 (15)	N10—C22—N13	117.4 (3)
Ca34—O2—H61^i^	100.9 (12)	N11—C22—N13	120.0 (3)
C32—O3—Ca34^iii^	83.2 (9)	C21—C23—C28	120.0 (4)
C14—N7—C17	114.3 (4)	C21—C23—H49	120.0 (10)
C14—N7—C19	115.5 (5)	C28—C23—H49	119.9 (10)
C17—N7—C19	121.8 (6)	C21—C24—C29	120.3 (3)
C15—N8—C18	120.1 (3)	C21—C24—H50	120.0 (11)
C15—N8—H35	120.0 (9)	C29—C24—H50	119.7 (12)
C18—N8—H35	120.0 (9)	N12—C25—C27	112.1 (3)
C16—N9—C21	119.3 (4)	N12—C25—C32	110.7 (3)
C16—N9—H36	120.0 (14)	C27—C25—C32	111.6 (5)
C21—N9—H36	120.7 (14)	N12—C25—H51	107.4 (11)
C18—N10—C22	117.7 (3)	C27—C25—H51	107.4 (13)
C18—N10—H37	120.0 (9)	C32—C25—H51	107.4 (13)
C22—N10—H37	122.3 (9)	C28—C26—C29	118.58 (17)
C20—N11—C22	120.0 (4)	C28—C26—C30	121.3 (4)
C25—N12—C30	121.4 (4)	C29—C26—C30	120.0 (4)
C25—N12—H38	120.0 (11)	C25—C27—C31	114.7 (4)
C30—N12—H38	118.5 (12)	C25—C27—H52	108.6 (15)
C22—N13—H39	109.4 (12)	C31—C27—H52	109 (3)
C22—N13—H40	110 (3)	C25—C27—H53	109.5 (16)
H39—N13—H40	109.5 (19)	C31—C27—H53	106.9 (14)
N7—C14—C15	108.57 (18)	H52—C27—H53	108.5 (18)
N7—C14—C16	110.57 (14)	C23—C28—C26	120.7 (3)
C15—C14—C16	110.9 (2)	C23—C28—H54	119.9 (11)
N7—C14—H41	108.9 (10)	C26—C28—H54	119.4 (12)
C15—C14—H41	108.9 (11)	C24—C29—C26	120.8 (3)
C16—C14—H41	108.9 (13)	C24—C29—H55	119.2 (14)
N8—C15—C14	109.7 (2)	C26—C29—H55	120.1 (14)
N8—C15—H42	109.4 (15)	O2—C30—N12	121.5 (3)
C14—C15—H42	109.4 (12)	O2—C30—C26	117.6 (3)
N8—C15—H43	109.5 (15)	N12—C30—C26	120.0 (5)
C14—C15—H43	109.4 (11)	C27—C31—C33	114.3 (5)
H42—C15—H43	109.4 (7)	C27—C31—H56	110 (3)
N9—C16—C14	112.9 (4)	C33—C31—H56	107.0 (13)
N9—C16—H44	108.9 (19)	C27—C31—H60	108.6 (12)
C14—C16—H44	108.9 (9)	C33—C31—H60	109 (2)
N9—C16—H45	109.5 (12)	H56—C31—H60	108.7 (15)
C14—C16—H45	107.8 (17)	O3—C32—O4	125.1 (4)
H44—C16—H45	108.9 (10)	O3—C32—C25	115.8 (4)
N7—C17—C18	117.6 (2)	O4—C32—C25	118.0 (3)
N7—C17—C20	121.7 (3)	O5—C33—O6	124.1 (4)
C18—C17—C20	119.76 (18)	O5—C33—C31	115.7 (4)
N8—C18—N10	116.4 (3)	O6—C33—C31	116.8 (4)
N8—C18—C17	118.6 (4)	N12—H38—Ca34	105.9 (10)
N10—C18—C17	119.3 (2)	O2—Ca34—O3^v^	144.4 (5)
N7—C19—H46	109.5 (17)	O2—Ca34—H38	69.2 (4)
N7—C19—H47	109.4 (11)	O3^v^—Ca34—H38	143.9 (6)
H46—C19—H47	110 (2)	O2—Ca34—O59	98.8 (5)
N7—C19—H48	109.4 (18)	O3^v^—Ca34—O59	116.4 (5)
H46—C19—H48	109.6 (15)	H38—Ca34—O59	38.4 (5)
H47—C19—H48	109 (2)	H61—O57—H62	142 (2)
O1—C20—N11	118.8 (3)	O2^i^—H61—O57	179.9 (13)
O1—C20—C17	124.4 (6)	Ca34—O59—H65	92.0 (12)
N11—C20—C17	113.3 (3)	Ca34—O59—H66	138.1 (17)
N9—C21—C23	121.0 (3)	H65—O59—H66	128 (2)
N9—C21—C24	119.5 (3)	H63—O58—H64	163 (4)
C23—C21—C24	119.41 (16)		

Symmetry codes: (i) −*x*+1, −*y*+1, −*z*+1; (ii) −*x*, *y*−1/2, −*z*+3/2; (iii) −*x*+1, *y*−1/2, −*z*+3/2; (iv) −*x*, *y*+1/2, −*z*+3/2; (v) −*x*+1, *y*+1/2, −*z*+3/2.

### Poly[[(µ-(2S)-2-{[4-({[(6S)-2-amino-5-methyl-1,4,5,6,7,8-hexahydropteridin-6-yl]methyl}amino)phenyl]formamido}pentanedioato)diaquacalcium(II)] monohydrate] (I_VASP) Crystal data

**Table:**

[Ca(C_20_H_23_N_7_O_6_)(H_2_O)_2_]·H_2_O	*c* = 15.4287 Å
*M**_r_* = 551.57	β = 117.86°
Monoclinic, *P*2_1_/*c*	*V* = 2532.84 Å^3^
*a* = 16.4610 Å	*Z* = 4
*b* = 11.2801 Å	*T* = 295 K

### Poly[[(µ-(2S)-2-{[4-({[(6S)-2-amino-5-methyl-1,4,5,6,7,8-hexahydropteridin-6-yl]methyl}amino)phenyl]formamido}pentanedioato)diaquacalcium(II)] monohydrate] (I_VASP) Data collection

**Table:**

*h* = →	*l* = →
*k* = →	

### Poly[[(µ-(2S)-2-{[4-({[(6S)-2-amino-5-methyl-1,4,5,6,7,8-hexahydropteridin-6-yl]methyl}amino)phenyl]formamido}pentanedioato)diaquacalcium(II)] monohydrate] (I_VASP) Fractional atomic coordinates and isotropic or equivalent isotropic displacement parameters (Å^2^)

**Table:**

	*x*	*y*	*z*	*B*_iso_*/*B*_eq_	
O1	0.13566	0.70774	1.00951		
O2	0.41966	0.42136	0.58093		
O3	0.29242	−0.00251	0.67295		
O4	0.42010	0.10694	0.75199		
O5	0.43639	−0.17961	0.69842		
O6	0.39155	−0.25179	0.54798		
N7	0.05880	0.68205	0.79615		
N8	−0.11769	0.58664	0.70651		
N9	0.09150	0.52271	0.67370		
N10	−0.12035	0.58077	0.85798		
N11	0.00194	0.64850	1.00439		
N12	0.39032	0.22700	0.58383		
N13	−0.12688	0.56526	1.00539		
C14	0.00480	0.69869	0.68938		
C15	−0.09826	0.67389	0.64879		
C16	0.05100	0.63726	0.63436		
C17	0.01069	0.66421	0.85097		
C18	−0.07577	0.61244	0.80519		
C19	0.13942	0.76049	0.83468		
C20	0.05307	0.67737	0.95737		
C21	0.15616	0.47466	0.65244		
C22	−0.07910	0.59947	0.95634		
C23	0.20736	0.37338	0.70253		
C24	0.17614	0.52626	0.58049		
C25	0.33511	0.12286	0.57521		
C26	0.29356	0.37675	0.60998		
C27	0.35398	0.02425	0.51743		
C28	0.27321	0.32543	0.68094		
C29	0.24465	0.47964	0.56231		
C30	0.37015	0.34014	0.59206		
C31	0.44264	−0.04915	0.57708		
C32	0.35019	0.07457	0.67412		
C33	0.42236	−0.16853	0.60989		
H35	−0.18561	0.56359	0.67704		
H36	0.09276	0.50005	0.73826		
H37	−0.18480	0.54237	0.82579		
H38	0.45058	0.21627	0.57917		
H39	−0.17049	0.49511	0.97330		
H40	−0.08332	0.55098	1.07772		
H41	0.00844	0.79356	0.67308		
H42	−0.13291	0.75868	0.64695		
H43	−0.12671	0.64234	0.57308		
H44	−0.00037	0.62877	0.55666		
H45	0.10382	0.69757	0.63470		
H46	0.11872	0.85487	0.82828		
H47	0.17518	0.74691	0.79055		
H48	0.18556	0.74050	0.91150		
H49	0.19554	0.33384	0.76029		
H50	0.14189	0.60725	0.54241		
H51	0.26219	0.14818	0.53432		
H52	0.35647	0.06664	0.45434		
H53	0.29437	−0.03566	0.48652		
H54	0.31436	0.25156	0.72465		
H55	0.26225	0.52604	0.51125		
H56	0.47397	−0.06874	0.52950		
H60	0.49337	0.00057	0.64075		
Ca34	0.57864	0.41693	0.68106		
O57	0.64519	0.54999	0.60875		
H61	0.62494	0.61942	0.63160		
H62	0.61835	0.55840	0.53548		
O59	0.57705	0.24688	0.59726		
H65	0.60649	0.18401	0.64869		
H66	0.59473	0.24136	0.54090		
O58	0.37907	0.88448	0.24975		
H63	0.32047	0.91148	0.19674		
H64	0.40245	0.82484	0.21898		

### Poly[[(µ-(2S)-2-{[4-({[(6S)-2-amino-5-methyl-1,4,5,6,7,8-hexahydropteridin-6-yl]methyl}amino)phenyl]formamido}pentanedioato)diaquacalcium(II)] monohydrate] (I_VASP) Hydrogen-bond geometry (Å, º)

**Table:**

*D*—H···*A*	*D*—H	H···*A*	*D*···*A*	*D*—H···*A*
N8—H35···O57^i^	1.02	2.48	3.490	169
N10—H37···O3^ii^	1.03	1.85	2.805	152
N12—H38···O59	1.03	2.00	2.991	160
N13—H39···O3^ii^	1.03	2.21	2.928	125
N13—H39···O1^iii^	1.03	2.34	3.086	128
O57—H61···O4^iv^	0.98	2.24	2.888	122
O57—H62···O2^v^	1.01	1.62	2.625	176
O58—H64···O5^vi^	1.00	1.81	2.749	156
O59—H65···O58^v^	1.00	1.66	2.588	151
O59—H66···O6^vii^	1.04	1.49	2.525	170
C16—H45···O1^viii^	1.10	2.47	3.349	136
C19—H48···O1	1.09	2.06	2.791	121
C24—H50···O1^viii^	1.09	2.14	3.157	154
C25—H51···N13^ix^	1.10	2.22	3.120	137
C28—H54···O4	1.09	2.28	3.263	150
C31—H60···O58^v^	1.10	2.37	3.446	165

Symmetry codes: (i) *x*−1, *y*, *z*; (ii) −*x*, *y*+1/2, −*z*+3/2; (iii) −*x*, −*y*+1, −*z*+2; (iv) −*x*+1, *y*+1/2, −*z*+3/2; (v) −*x*+1, −*y*+1, −*z*+1; (vi) *x*, −*y*+1/2, *z*−1/2; (vii) −*x*+1, −*y*, −*z*+1; (viii) *x*, −*y*+3/2, *z*−1/2; (ix) −*x*, *y*−1/2, −*z*+3/2.
